# Intraocular lens power calculation after radical keratotomy and photorefractive keratectomy: A case report

**DOI:** 10.1097/MD.0000000000029465

**Published:** 2022-07-08

**Authors:** Tianxu Xiong, Jiancheng Mu, Hao Chen, Wei Fan

**Affiliations:** Department of Ophthalmology, West China Hospital of Sichuan University, Chengdu, Sichuan Province, China.

**Keywords:** cataract, intraocular lens power, corneal refractive surgery, high myopic

## Abstract

**Rationale:**

To report a rare case of calculating the IOL power in a cataract patient who underwent both radial keratotomy (RK) and photorefractive keratectomy (PRK).

**Patient concerns:**

A 48-year-old woman underwent bilateral RK at age 22 and bilateral PRK at age 46. She developed bilateral corneal haze and corneal endothelial inflammation and received steroids therapy for long time after PRK. Then she was referred to our hospital due to decreased vision in the both eyes.

**Diagnoses:**

The patient was diagnosed with binocular complicated cataract, corneal haze, high myopia and post corneal refractive surgery (RK and PRK).

**Interventions:**

The patient underwent bilateral phacoemulsification. The IOL power was calculated using SRK/T formula for RK and Haigis-L formula for PRK, respectively. We finally selected the Haigis-L formula and the intraocular lens (SN60WF) was implanted within the capsular bag.

**Outcomes:**

After the surgery, both eyes showed myopia drift, and the right eye continuously fluctuated in refractive results. However, by nearly 1 year later, refractive results in both eyes had stabilized, and no other complications had occurred.

**Lessons:**

IOL power in patients who undergo both RK and PRK can be reliably calculated using the Shammas-PL, Average of multiple formulas, or Barret True-K No History formulas. Haigis-L formula is not suitable. Such patients require at least three months after surgery to attain refractive stability in both eyes.

## 1. Introduction

Refractive surgery is widely used to improve vision, but many patients gradually develop cataract after corneal refractive surgeries such as radial keratotomy (RK), excimer laser photorefractive keratectomy (PRK), or laser-assisted in situ keratomileusis (LASIK). To take into account the needs of patients with prior corneal refractive surgery, several studies have focused on the accurate calculation of intraocular lens (IOL) power.^[1–3]^ However, there is no perfect formula for these patients until now. According to a recent review,^[4]^ for myopic-LASIK/PRK eyes, in most studies best outcomes did not exceed 75% accuracy within 0.5 D of target, the accuracy is even lower for RK eyes. Therefore, it is more unpredictable for patients who received 2 corneal refractive surgeries. So far, we are aware of only 1 study reporting the accurate calculation of IOL power in patients who have undergone 2 types of corneal refractive surgery, which in that case were RK and LASIK.^[5]^

Therefore, in the present study, we describe how we calculated the IOL power in a cataract patient who previously underwent both RK and PRK, and we report on postoperative refractive error and refractive state.

## 2. Case Presentation

A 48-year-old woman with previous RK and PRK was referred to our hospital because of gradually reduced binocular vision. The patient had undergone bilateral RK at the age of 22 at a local private hospital. Prior to RK surgery, her binocular myopic diopter was about −6.0 D, and her postoperative visual acuity was good. Unfortunately, the detailed pre- or postoperative data for RK were not available, but all of the following diagnosis and treatment in other hospitals came from the patient’s medical records.

At the age of 46, she was admitted to another local hospital because of myopic regression, with an uncorrected visual acuity of 0.1 in both eyes and a best corrected visual acuity (BCVA) of −4.50DS/−1.50DC*80→0.5 in the right eye and −4.50DS/−1.00DC*100→0.7 in the left eye. The central corneal thickness was 652 μm (right eye) and 649 μm (left eye), and the corresponding intraocular pressure was 25.7 and 26 mm Hg. Corneal endothelial cell count was normal in both eyes. She underwent bilateral enhanced PRK at that hospital.

At 42 days after PRK, her right eye developed corneal haze (1.5 degrees) and its uncorrected visual acuity decreased to 0.07, while the left eye showed uncorrected visual acuity of 0.6. The patient received prednisolone as an antiinflammatory treatment for her right eye, but her condition worsened, and her both eyes developed corneal edema with folds of Descemet Membrane.

Two months after PRK, her corneal edema and haze (right eye 3 degrees, left eye 2 degrees) became worse, and she was referred to a hospital in Beijing, where she was diagnosed with binocular corneal dermatitis caused by herpes simplex virus because the Torch examination (Toxoplasmosis, other infections, Rubella, Cytomegalovirus, and Herpes simplex) showing herpes simplex virus infection positive. She was treated with oral and local antiinflammatory and antiviral drugs for next 15 months, which improved and stabilized her bilateral corneal condition (right eye 2 degrees, left eye basically transparent) and BCVA (right eye −8.00DS→0.4, left eye −3.75DS→0.6).

**Figure 1.**
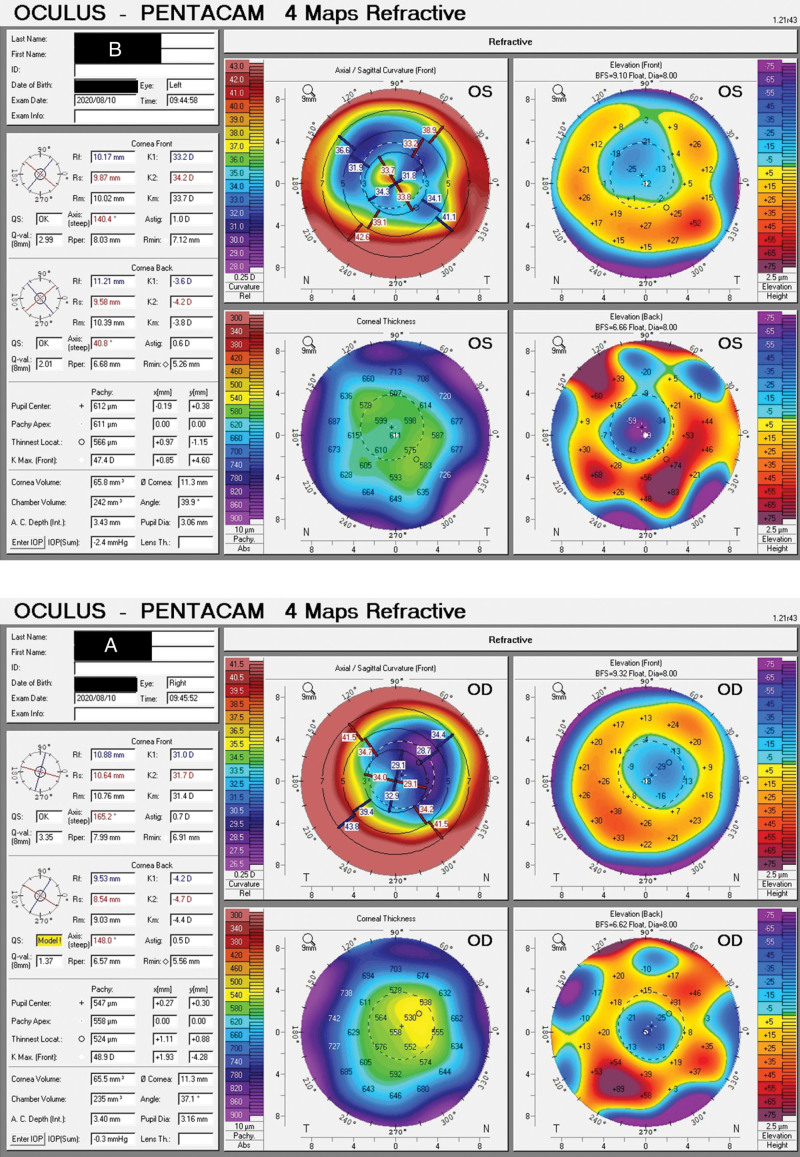
Pentacam 4 maps refractive of the right eye (A) and left eye (B).

At the age of 48, the patient again visited the hospital where she had PRK because of progressive deterioration of vision. She was diagnosed with bilateral complicated cataract and corneal haze (right eye 2 degrees and left eye 1 degree) and was then referred to our hospital for cataract surgery. Ophthalmic examination showed uncorrected visual acuity of 0.04 in her right eye and 0.08 in her left eye, while the corresponding BCVA values were −8.00DS/−1.25DC*90 →0.05 and −11.00DS/−1.50DC*45 →0.2. In addition, the patient showed 16 radial scars; central corneal haze, which was more severe in the right eye; and lens opacity in both eyes, scored as C2N3P3 in the right eye and C2N2P2 in the left eye according to the Lens Opacities Classification System III. Corneal endothelial cells could not be counted. Fundus examination using optical coherence tomography (OCT III, Carl Zeiss Meditec AG, Jena, Germany) revealed changes typical of high myopia in both eyes, with slight splitting in the macular area. Keratometry and biometry data were measured using the Zeiss IOL Master 700 system (Carl Zeiss Meditec AG, Jena, Germany) and Pentacam system (Oculus, Wetzlar, Germany) (Fig. 1 A, B) (Table 1). Corneal haze was measured using cornea densitometry in Pentacam system (Oculus, Wetzlar, Germany) (Table 2).

**Table 1:** Preoperative biometric data.

Measurement system		Right eye	Left eye
IOL Master 700	Axial length (mm)	30.95	30.68
	Anterior chamber depth (mm)	3.89	3.96
	Lens thickness (mm)	3.77	3.83
	White to white (mm)	11.5	11.9
	Spherical equivalence (D)	31.95	33.35
	K1 (D)	31.75@53°	32.78@28°
	K2 (D)	32.15@143°	33.93@118°
Pentacam - anterior corneal surface	K1	31.1	33.2
	K2	31.5	34.2
	Km	31.3	33.7
	Steep axis	176.2	140.4
	astigmatism	0.4	1.0
Pentacam - posterior corneal surface	K1	–4.4	–3.6
	K2	–4.6	–4.2
	Km	–4.5	–3.8
	Axis (steep)	157.7	40.8
	astigmatism	0.2	0.6
Corneal topography	Ks	32.55@8°	34.20@96°
	Kf	31.91@98°	33.70@6°
	astigmatism	0.64	0.49

**Table 2:** Cornea densitometry annulus and layer averages.

		0–2 mm	2–6 mm	6–10 mm	10–12 mm	Total
Anterior (120 μm)	Right eye	66.1	47.1	26.5	37.4	42.4
	Left eye	34.9	34.8	26.2	46.0	32.6
Center layer	Right eye	27.9	24.1	18.8	28.2	23.0
	Left eye	14.6	18.6	18.7	25.6	18.5
Posterior (60 μm)	Right eye	15.9	14.5	14.9	18.7	15.1
	Left eye	10.7	11.7	14.7	15.5	12.9
Total	Right eye	36.7	28.5	20.1	28.1	26.8
	Left eye	20.1	21.7	19.9	29.0	21.4

The patient’s right eye had worse postoperative visual acuity than the left eye due to more severe haze. To reduce the postoperative refractive error and improve patient’s vision, we first performed cataract surgery on the right eye. Considering the patient’s habits and the needs of near vision, we set the target diopter of the right eye as −3.0 D. We calculated the IOL power using SRK/T formula for postRK condition and Haigis-L formula for postPRK condition, respectively, based on our experiences and literatures^[4,6]^ that these formulas were relatively accurate in any single corneal surgical situation. However, quite different values for the selected IOL (SN60WF, AcrySof IQ, Alcon Laboratories, Inc., Fort Worth, USA) were obtained: 19.0 D for postRK condition, 27.5 D for postPRK condition. Given that the Haigis-L formula has been used to determine IOL power after myopic RK and LASIK,^[5]^ we implanted the 27.5 D IOL in the patient’s right eye. On the second postoperative day, the uncorrected visual acuity is 0.08 in her right eye and the corresponding BCVA values were −6.00DS→0.3, which showed a myopic shift of 3.0D. Hence, the target diopter of the left eye was set to 0.0D, and a SN60WF 21.5D intraocular lens was implanted. The surgery was challenging because of the corneal radial scars, high axial myopia [axial length (AL) >30 mm], uncountable corneal endothelial cells, and corneal haze. Nevertheless, we were able to perform bilateral cataract phacoemulsification and intraocular lens implantation uneventfully at low intraoperative perfusion pressure.

**Figure 2.**
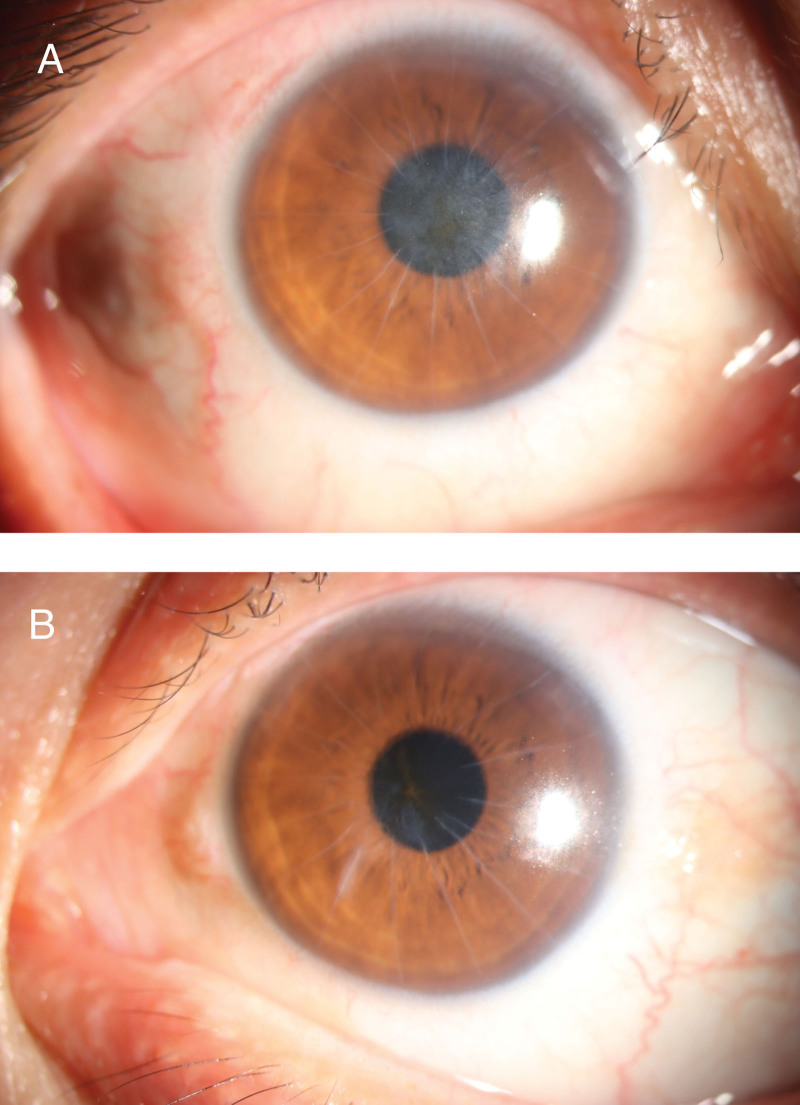
Postoperative anterior segment photographs of the right eye (A) and left eye (B).

After surgery, the patient was treated with tobramycin and dexamethasone eyedrops and diclofenac sodium eyedrops. Tobramycin and dexamethasone eyedrops were applied 4 times a day in the first week, followed by 3 times a day in the second week, 2 times a day the third week and 1 time a day the fourth week. In addition, diclofenac sodium eyedrops were applied 4 times a day for 6 weeks under close follow-ups. The binocular spherical equivalent changed continuously during postoperative follow-up, stabilizing more than 3 months after surgery (Table 3). By 3 months after surgery, intraocular pressure had increased slightly (right eye, 15.9 mm Hg; left eye, 22.6 mm Hg), and pressure was even higher after 6 months (right eye, 41.6 mm Hg; left eye, 30.6 mm Hg). The patient was given local pressure-lowering drugs, and by 8 months after the operation, intraocular pressure had returned to normal (right eye, 13.3 mm Hg; left eye, 15.6 mm Hg), the left eye showed stable refractive status, but the right eye showed a −1.5 D more myopic shift, reaching −8.5 D. At 11 months after the operation, the intraocular pressure was still normal (right eye, 10.5 mm Hg; left eye, 13.3 mm Hg), the left eye still showed stable refractive status, while the right eye had returned to the same refractive status as at 6 months after surgery, which was −7.0 D. Corneal topography and biological measurements using the IOL Master and Pentacam systems showed no significant variation in ocular axis, anterior chamber depth, or corneal curvature at 6, 8, or 11 months after the operation (Table 4). Besides, there were no other complications had occurred during postoperative follow-up (Fig. 2 A, B).

**Table 3:** Optometry results of postoperative follow-up.

	Right eye		Left eye	
Time after operation	Optometry results	Spherical equivalence	Optometry results	Spherical equivalence
1 week	–4.00DS/ –1.00DC* 65	–4.50	–1.00DS/ –0.50DC* 80	–1.25
1 month	–4.25DS/ –1.50DC* 65	–5.00	–0.50DC * 100	–0.25
3 months	–5.75DS/ –1.00DC * 70	–6.25	–0.25DS/ –1.50DC * 60	–1.00
3.5 months	–7.00DS/ –0.75DC * 80	–7.375	–0.50DS/ –1.50DC * 90	–1.25
6 months	–7.00DS/ –0.75DC * 90	–7.375	–0.50DS/ –1.50DC * 90	–1.25
8 months	–8.50DS/ –0.75DC * 80	–8.875	–0.50DS/ –1.50DC * 120	–1.25
11 months	–7.00DS	–7.00	–1.00DS/ –1.00DC * 100	–1.50

**Table 4:** Optometry measurements at 6, 8, and 11 months postoperation.

		6 months		8 months		11 months	
Measurement system or parameter		Right eye	Left eye	Right eye	Left eye	Right eye	Left eye
Vision	uncorrected	0.06	0.5	0.1	0.4	0.06	0.5
	Best corrected	0.5	0.9	0.3	0.7	0.5	0.6
Intraocular pressure (mm Hg)		41.6	30.6	13.3	15.6	10.5	13.3
IOL Master 700	Axial length (mm)	30.98	30.68	31.02	30.71	31.04	30.72
	Anterior chamber depth (mm)	4.8	4.89	4.78	4.86	4.73	4.82
	White to white (mm)	11.4	11.6	11.6	11.8	11.6	11.7
	K1	32.54@58	33.08@22	32.63@65	33.48@29	31.73@74	33.10@30
	K2	32.83@148	33.94@112	33.47@155	34.14@119	33.60@164	33.81@120
	ΔK	–0.28	–0.86	–0.84	–0.67	–1.86	–0.71
Pentacam–Anterior corneal surface	K1	31	34.6	31.8	33.9	31.3	34.0
	K2	32	34.9	32.8	34.7	32.3	35.3
	Km	31.5	34.8	32.3	34.3	31.8	34.6
	Axis (steep)	147.8	174.1	176.9	9.5	151.3	167.4
	astigmatism	1.1	0.3	1.0	0.7	1.1	1.3
Pentacam- Posterior corneal surface	K1	–4.6	–4.1	–4.5	–4.3	–4.5	–3.9
	K2	–5	–4.5	–4.8	–4.6	–4.9	–4.3
	Km	–4.8	–4.3	–4.6	–4.5	–4.7	–4.1
	Axis (steep)	175.7	55.4	161.2	70.8	154.2	33.4
	astigmatism	0.4	0.4	0.3	0.3	0.5	0.3
Corneal topography	Ks	33.35@8	34.37@87	33.96@11	34.92@80	34.02@3	34.64@91
	Kf	32.64@98	34.06@177	33.07@101	34.51@170	32.83@93	34.36@1
	astigmatism	0.72	0.31	0.89	0.42	1.19	0.28

At 11 months after the operation, the patient’s right eye with a SN60WF 27.5 D intraocular lens had an actual spherical equivalent of −7.0 D, a target diopter of −3.0 D, and a refractive error of −4.0 D. The patient’s left eye with an IQ 21.5 D intraocular lens had an actual spherical equivalent of −1.5 D, a target diopter of 0.0 D, and a refractive error of −1.5 D.

Given these postoperative refractive errors, we retrospectively entered all of the preoperative biometric parameters into the website of the American Society of Cataract and Refractive Surgery (ASCRS) to analyze and compare the IOL power for postRK condition using IOL Master, Pentacam, Barrett True K formulas, the Average of multiple formulas, and for postPRK condition using Shammas-PL, Haigis-L, Pentacam, Barrett True K No History formulas, and the Average of multiple formulas.^[7]^ The differences are shown in Table 5. As can be seen, Shammas-PL, the Average of multiple formulas, and Barret True-K No History formulas were reasonably accurate, while Haigis-L produced large errors.

**Table 5:** Intraocular lens power calculated using formulas from ASCRS online calculator and analysis.

	Preoperative target		Actual refraction				Postoperative analysis		
Right eye	Target refraction		Postoperative refraction	Error	Preoperative formula	IOL suggestion	Condition	Formula	IOL suggestion
	–3		–7	–4	Haigis-L	21	post RK	IOL Master/Lenstar	20.77
								Pantacam	21.59
								Barrett True K	23.13
								Average	21.83
								SRK-T	21.83
							Post myopic PRK	Shammas	23.95
								Haigis-L	27.49
								Pantacam	22.23
								Barrett True K No history	24.85
								Average	24.63
	**Preoperative target**		**Actual refraction**				**Postoperative analysis**		
**Left eye**	**Planned target refraction**	**Corrected target refraction**	**Postoperative refraction**	**Error**	**Preoperative formula**	**IOL suggestion**	**Condition**	**Formula**	**IOL suggestion**
	–3	0	–1.5	–1.5	Haigis-L	21	post RK	IOL Master/Lenstar	15.47
								Pantacam	15.29
								Barrett True K	17.73
								Average	16.16
							Post myopic LASIK	Shammas	18.87
								Haigis-L	21.04
								Pantacam	16.18
								Barrett True K No History	19.44
								Average	18.89

## 3. Discussion

Cataract surgery and accurate IOL power prediction in patients with prior surgeries of both RK and PRK remain challenging due to the postoperative risk of radial corneal incision, central corneal opacity, high axial myopia and change in corneal curvature. Here we used the SRK/T and Haigis-L formulas to calculate the IOL power in the rare case of a cataract patient who had undergone both RK and PRK. Although our approach was based on a recent report of a patient who had undergone RK and LASIK,^[5]^ the IOL power in our patient was remarkably different after RK and PRK, and her right eye showed large postoperative refractive error.

The Haigis-L formula has been identified as one of the most accurate formulas for predicting IOL power in patients who have undergone both RK and LASIK.^[5]^ However, there was a big difference between the SRK/T formula for postRK condition and Haigis-L formula for postPRK condition in calculating the IOL power in our case. The 2 procedures exert different effects on the cornea. RK flattens the central cornea and steepens the peripheral cornea, it changes the corneal curvature by deforming the whole cornea without any tissue loss.^[8]^ In contrast, PRK reduces corneal curvature due to tissue removal from the central area of the anterior corneal surface.^[9,10]^ Our lack of medical history about the present patient prevented us from determining the effects of PRK and RK on corneal curvature in the 4.0-mm central area. In addition, our patient underwent RK nearly 30 years before we examined her, and various factors during that time may have affected corneal curvature. Patients who previously underwent corneal refractive surgery often develop refractive errors after cataract surgery, especially hyperopia drift.^[7]^ Our patient would prefer myopia to hyperopia in the event of refractive error. Therefore, we set the target diopter to −3.0 D (myopia) to counteract the possible hyperopic drift and used the Haigis-L formula to preoperatively calculate the IOL power based on postPRK condition alone.

Various formulas can be used to calculate IOL power in eyes that have undergone PRK, including Haigis-L, Barret True-K No History and Shammas-PL. However, the accuracy of each formula remains controversial. Barret True-K No History has recently been identified as the most accurate,^[11]^ and other meta-analyses have identified Barret True-K No History, the Average of multiple formulas, OCT (optical coherence tomography), and ORA (optiwave refractive analysis)^[12,13]^ as the most reliable formulas for accurate IOL power prediction. However, the applicability of those findings to our patient is unclear given that the ocular axis in most of those studies was up to 30 mm. Comparison of IOL power formulas according to AL suggested that Shammas-PL provides the most accurate results when the ocular axis is longer than 30 mm,^[14]^ while Shammas-PL, SRK/T_corrected K_, and Barret True-K No History formulas are more accurate than Haigis-L when AL is longer than 29 mm and corneal curvature is <35 D.^[15]^ The Haigis-L, Shammas-PL, and Barret True-K No History formulas performed well in a patient who had undergone both RK and LASIK^[5]^ and whose ocular AL was 27.37 mm, minimum corneal curvature (K1) was 35.83 D, and maximum corneal curvature (K2) was 36.33 D. In our case, the ocular AL was longer than 30 mm and the corneal curvature was lower than 35 D, implying that Shammas-PL, Average, Barret True-K No History could accurately predict the IOL power, whereas the Haigis-L formula would be unsuitable. Based on these findings, we conclude that when the eye axis is longer than 30 mm and the corneal curvature is low (K < 35 D), the Shammas-PL, Average, and Barret True-K No History formulas can accurately calculate the IOL power in cataract patients who have previously undergone both RK and PRK.

A refractive error of −4.0 D was observed in the right eye and −1.5 D in the left eye, with the right eye showing less refractive stability than the left eye. It is noteworthy that the corneal curvature of our patient’s right eye was lower than that of the left eye by 2.0 D, and the average cornea densitometry between 0-2 mm and 2-6 mm of our patient’s right eye was higher than that of the left eye by 16.6 and 6.8, which could explain the greater refractive error in her right eye. Besides, the more obvious central corneal haze in her right eye may change the refractive properties of the cornea, which can distort measurements and thereby reduce the accuracy of IOL power calculation. For the calculation of IOL power in the second eye (left), we assumed that the left eye would produce the same refractive error as the right eye, so the target of 0.0 D was set which ended up with −1.5 D postoperatively. Zhang et al^[16]^ reported that it may be more accurate to estimate the degree of error in the second eye as half the refractive error in the first eye. Consistent with that report, our patient had a myopia drift of 4.0 D in her right eye and 1.5 D in her left eye.

A possible explanation for the postoperative refractive fluctuation could be the morning-to-evening change in ophthalmic measurements after RK, which can persist for more than 11 years, although the amount of this change is small in most patients. In some individuals, this diurnal fluctuation may be a permanent sequela of RK.^[17,18]^ We measured all the biometry and optometry data between 9:00 am to 12:00 am in this case to minimize the influence of this diurnal changes. In addition, the possible effect of surgery on biometric parameters was also taken into account. However, there was no significant difference in the pre- and postoperative biometric data in ocular AL, anterior chamber depth, lens thickness, or white-to-white corneal diameter. Finally, the same optometrist finished all optometry after operation for this patient and the refractive results of the right eye fluctuated considerably compared to the left eye. Thus, it is possible that the serious corneal haze in the right eye affect the accuracy of the optometrist even at 8 months postsurgery.

Even with the most advanced biometry measurement devices and IOL calculation formulas, for myopic-LASIK/PRK eyes, the best outcomes in most studies did not exceed 75% accuracy within 0.5 D of target and the accuracy is even lower for eyes with previous RK.^[4]^ To improve the accuracy of IOL power calculation results, intraoperative wavefront aberrometry^[19,20]^ and postoperative IOL adjustment^[21,22]^ also show promising results. Doctors can consider these methods when their hospitals have these devices available. Besides, Doctor-patient communication is also particularly important,^[4,23]^ so that patients can understand the possibility of postoperative refractive error and have reasonable expectations about the surgical outcome.

Our findings conclude that the Shammas-PL, the Average of multiple formulas, and Barret True-K No History formulas can provide accurate IOL power results based on postPRK condition alone in cataract patients who have undergone 2 types of refractive surgery. In contrast, the Haigis-L formula may be unsuitable when the ocular axis is long (>30 mm) and corneal curvature is low (<35 D), as it produces large refractive errors. Therefore, the surgeon should operate first on the eye with poor predicted postoperative vision and adjust the IOL power for the other eye according to the refractive results of the first eye after cataract surgery. Since the present study involved only 1 patient, additional research is needed to identify the best methods for calculating IOL power in patients who have undergone 2 kinds of corneal refractive surgery.

## Author contributions

Conceptualization: Tianxu Xiong, Wei Fan.

Data curation: Tianxu Xiong, Jiancheng Mu.

Investigation: Hao Chen.

Methodology: Tianxu Xiong, Wei Fan.

Writing original draft: Tianxu Xiong, Jiancheng Mu.

Writing review & editing: Wei Fan.
